# The lncRNA HOTAIR regulates autophagy and affects lipopolysaccharide‐induced acute lung injury through the miR‐17‐5p/*ATG2/ATG7/ATG16* axis

**DOI:** 10.1111/jcmm.16737

**Published:** 2021-06-27

**Authors:** Yujun Li, Zhike Liang, Hua He, Xiaomei Huang, Zexun Mo, Jinwen Tan, Weihong Guo, Ziwen Zhao, Shuquan Wei

**Affiliations:** ^1^ Department of Pulmonary and Critical Care Medicine Guangzhou First People’s Hospital School of Medicine South China University of Technology Guangzhou China

**Keywords:** acute lung injury, apoptosis, autophagy, lncRNA HOTAIR, miR‐17‐5p

## Abstract

Long non‐coding ribonucleic acids (lncRNAs) play critical roles in acute lung injury (ALI). We aimed to explore the involvement of lncRNA HOX transcript antisense intergenic ribonucleic acid (HOTAIR) in regulating autophagy in lipopolysaccharide (LPS)‐induced ALI. We obtained 1289 differentially expressed lncRNAs or messenger RNAs (mRNAs) via microarray analysis. HOTAIR was significantly upregulated in the LPS stimulation experimental group. HOTAIR knockdown (si‐HOTAIR) promoted cell proliferation in LPS‐stimulated A549 and BEAS‐2B cells, suppressing the protein expression of autophagy marker light chain 3B and Beclin‐1. Inhibition of HOTAIR suppressed LPS‐induced cell autophagy, apoptosis and arrested cells in the G0/G1 phase prior to S phase entry. Further, si‐HOTAIR alleviated LPS‐induced lung injury in vivo. We predicted the micro‐ribonucleic acid miR‐17‐5p to target HOTAIR and confirmed this via RNA pull‐down and dual luciferase reporter assays. miR‐17‐5p inhibitor treatment reversed the HOTAIR‐mediated effects on autophagy, apoptosis, cell proliferation and cell cycle. Finally, we predicted autophagy‐related genes (ATGs) *ATG2*, *ATG7* and *ATG16* as targets of miR‐17‐5p, which reversed their HOTAIR‐mediated protein upregulation in LPS‐stimulated A549 and BEAS‐2B cells. Taken together, our results indicate that HOTAIR regulated apoptosis, the cell cycle, proliferation and autophagy through the miR‐17‐5p/*ATG2*/*ATG7*/*ATG16* axis, thus driving LPS‐induced ALI.

## 1 INTRODUCTION

Acute lung injury (ALI) is a life‐threatening respiratory disorder with high morbidity and mortality rates for which there is a notable lack of US Food and Drug Administration (FDA)‐approved drug therapies.^1^, ^2^ Lipopolysaccharides (LPS) are cell wall components of Gram‐negative bacteria that induce apoptosis in alveolar epithelial cells (ECs), such as A549 or bronchial epithelium transformed with Ad12‐SV40 2B (BEAS‐2B) cells. Therefore, LPS‐stimulated A549 and BEAS‐2B cells have emerged as clinically relevant models of ALI.^3^, ^4^

Autophagy is an evolutionarily conserved degradation pathway responsible for delivering cytoplasmic components to the lysosome in vesicles called autophagosomes.^5^ Autophagosome formation depends on several autophagy‐related genes (ATGs), including light chain 3B (*LC3B*) and *Beclin‐1*.^6^ Autophagy inhibition is known to ameliorate LPS‐induced ALI. For instance, Fu *et al* ^7^ found that hydrogen‐rich saline inhibited both LPS‐induced ALI and endothelial dysfunction by regulating autophagy. Likewise, Chen *et al* ^8^ reported that miR‐100 from microvesicles enhanced autophagy and ameliorated ALI. These studies indicate that autophagy is a potential therapeutic target in ALI, warranting further investigation.

Micro‐ribonucleic acids (miRNAs) are a class of small non‐coding RNAs (ncRNAs) that are ~22 nucleotides (nt) long and regulate messenger (mRNA) as well as long non‐coding RNA (lncRNA) expression at the post‐transcriptional level via miRNA binding sites.^9^, ^10^ MiRNAs, the study of which has become a research hotspot within molecular biology, are reported to play important regulatory roles in ALI pathogenesis, progression and treatment.^11^, ^12^ For instance, Neudecker *et al* ^13^ found that the transfer of miR‐223 from neutrophils to lung ECs dampens ALI in mice. Jansing *et al* ^12^ reported that miR‐21‐KO alleviates alveolar structure remodelling and inflammatory signalling in ALI. These studies suggest that miRNAs may serve as promising targets for the prevention and treatment of ALI. Even though miRNAs have been studied for decades, those involved in autophagy regulation have only recently received attention. Zhou *et al* ^14^ observed that mesenchymal stem cells could alleviate LPS‐induced ALI in mice via miR‐142a‐5p‐regulated pulmonary EC autophagy. Therefore, our current study aimed to explore novel miRNAs that induce autophagy in ALI.

LncRNAs are defined as transcripts of >200 nt in length without protein‐coding potential. Further, these can alter miRNA expression by acting as competing endogenous RNAs and can interact with translation machinery by targeting mRNA.^15^, ^16^ Different researchers have reported lncRNAs to have a variable influence on ALI over the past few years. Wang *et al* ^17^ found that lncRNAs were significantly altered in LPS‐induced ALI and that targeting lncRNA could suppress the LPS‐induced inflammatory response. Liao *et al* ^18^ found that lncRNA maternally expressed gene 3 (*MEG3*) could adsorb miRNA‐7B to regulate nucleotide‐binding oligomerization domain as well as leucine rich repeat and pyrin domain‐containing 3, thus suppressing LPS‐induced ALI. Studies have shown that lncRNAs can inhibit downstream‐related signal transduction through the miRNA/mRNA axis, reduce cell autophagy and alleviate ALI.^19^, ^20^ However, the exact mechanism through which lncRNAs regulate autophagy to induce ALI via the adsorption of miRNAs remains unknown.

In this study, we assessed the biological function of lncRNA HOTAIR and miR‐17‐5p as well as their effects on cell proliferation and apoptosis. Furthermore, we explored the regulatory network involving HOTAIR, miR‐17‐5p and autophagy to open new avenues for the treatment and diagnosis of ALI.

## 2 MATERIALS AND METHODS

### 2.1 Microarray analysis of differentially expressed miRNAs

We downloaded raw gene expression data from the US National Center for Biotechnology Information (NCBI) Gene Expression Omnibus (GEO; https://www.ncbi.nlm.nih.gov/geo/). The samples (filename GSE40885_RAW.tar) were divided into two groups: seven alveolar macrophages from lung subsegments treated with LPS (GSM1004102, GSM1004104, GSM1004106, GSM1004108, GSM1004110, GSM1004112 and GSM1004114) and seven alveolar macrophages from lung subsegments instilled with saline solution (GSM1004101, GSM1004103, GSM1004105, GSM1004107, GSM1004109, GSM1004111 and GSM1004113). We analysed the Affymetrix Human Genome U133 Plus Version 2.0 Array (GPL570) using the Affymetrix Transcriptome Analysis Console (both from Affymetrix). Differentially expressed lncRNAs/mRNAs were identified as having *P* < .05 and |fold change (FC)| > 2. We drew a heatmap and a volcano plot using the results of differentially expressed mRNA analysis.

### 2.2 Pathway enrichment analysis

We performed pathway enrichment analysis on the differentially expressed mRNAs using the Kyoto Encyclopedia of Genes and Genomes (KEGG) and the R software package clusterProfiler version 3.10.1 (https://guangchuangyu.github.io/software/clusterProfiler/).

### 2.3 Animals

All animal experiments were approved by the Animal Care and Use Committee of the First People’s Hospital of Guangzhou City, China, and conducted according to US National Institutes of Health (NIH) guidelines. The characteristics of experimental mice used in this study were as follows: genotype, C57BL/6; phenotype, specific‐pathogen‐free (SPF); body weight, 20‐30 g; sex, male; license, No. SCXK (Guangdong) 2016‐0041.

### 2.4 ALI mouse model

We randomly divided 24 C57BL/6 mice into four groups: a sham operation group, a model group, a lentivirus (LV)‐control group and a LV‐si‐HOTAIR group (n = 6 per group). To establish the ALI model, we anaesthetized mice with intraperitoneal injections of 1% pentobarbital sodium (50 mg/kg). Mice were endotracheally intubated with an indwelling needle. Using a 1‐mL syringe, we pushed 10 μg LPS in 50 μL phosphate‐buffered saline (PBS) into the tube. The sham group received an equal volume of PBS.^4^ We injected control and si‐HOTAIR lentiviruses (2 × 10^8^ TU/mL; Hanbio) through the tail vein 30 minutes before LPS stimulation. After 6 hours of stimulation, mice were killed, and lung tissues were removed and stored at −80°C.

### 2.5 Haematoxylin and eosin (H&E) staining

We placed lung tissue in 10% formalin overnight, dehydrated it and embedded it in paraffin. The tissue was sliced into 5‐mm thick sections, fixed on a glass slide, dried and then dyed using HE staining solution (Solarbio) as per the manufacturer’s instructions. We soaked the slices in xylene, in gradient‐concentration ethanol and then in haematoxylin before sealing them with resin. After drying, we observed changes to the alveolae and alveolar interstitial structure in lung tissue sections, photographing them under a light microscope.

### 2.6 RNA pull‐down assay

Biotinylated HOTAIR (Bio‐HOTAIR), miR‐17‐5p (Bio‐miR‐17‐5p), HOTAIR Mut (Bio‐HOTAIR‐Mut), miR‐17‐5p Mut (Bio‐miR‐17‐5p‐Mut) and their negative control (Bio‐NC or Bio‐miR‐NC) (Sangon Biotech Co., Ltd.) were transfected into the A549 and BEAS‐2B cells. Following incubation for 24 hours, the transfected cells were lysed, collected and incubated with Dynabeads M‐280 Streptavidin (Invitrogen; Thermo Fisher Scientific, Inc) for 10 minutes in 4°C. The bound RNAs were then subjected to RT‐qPCR for quantification and analysis as described above.

### 2.7 Cell culture

We purchased A549 (Cat. No. CCL‐185), BEAS‐2B (Cat. No. CRL‐9609) and 293T (Cat. No. CRL‐11268) cells as authenticated stocks from the American Type Culture Collection (ATCC). Both cell lines were maintained in Dulbecco’s modified Eagle’s medium supplemented with 10% foetal bovine serum (FBS) at 37°C and 5% CO_2_. Cells were tested for mycoplasma contamination approximately once a month using a MycoAlert Mycoplasma Detection Kit (Cat. No. LT07‐218; Lonza Cologne GmBH).

### 2.8 Transmission electron microscopy

We observed autophagy in A549 and BEAS‐2B cells under a transmission electron microscope. Cells from each experimental group were collected, digested with 2.5 g/L trypsin, centrifuged at 1000 *g*, washed with PBS, and collected in microcentrifuge tubes. We then fixed the cells with 25 g/L glutaraldehyde plus 10 g/L citric acid. After dehydration with graded ethanol and infiltration, cells were embedded in epoxy resin. We sliced the resin using an ultramicrotome, stained the cells with uranyl acetate as well as lead citrate and observed them via transmission electron microscopy (TEM).

### 2.9 Real‐time quantitative reverse transcription polymerase chain reaction (RT‐qPCR)

We extracted total RNA using TRIzol reagent (Invitrogen). The extracted RNA was reverse‐transcribed into complementary deoxyribonucleic acid (cDNA) using a PrimeScript RT Reagent Kit (TaKaRa) as per the manufacturer’s instructions. We performed RT‐qPCR using an ABI 7500 system (Applied Biosystems) and a SYBR Premix ExTaq II kit (TaKaRa). The primers for HOTAIR, glyceraldehyde 3‐phosphate dehydrogenase (*GAPDH*), miR‐17‐5p, and U6 were as follows: HOTAIR: forward, 5′‐CAGTGGGGAACTCTGACTCG‐3′, reverse, 5′‐GTGCCTGGTGCTCTCTTACC‐3′; *GAPDH*: forward, 5′‐GCTCATTTGCAGGGGGGAG‐3′, reverse, 5′‐GTTGGTGGTGCAGGAGGCA‐3′; miR‐17‐5p: forward, 5′‐ACACTCCAGCTGGGCAAAGTGCTTACAGTGC‐3′, reverse, 5′‐CTCAACTGGTGTCGTGGA‐3′; and U6: forward, 5′‐CTCGCTTCGGCAGCACA‐3′, reverse, 5′‐AACGCTTCACGAATTTGCGT‐3′. Relative HOTAIR and miR‐17‐5p expression levels were calculated via the $2^{‐\Delta \Delta C_{t}}$ method. We performed PCR experiments for each sample in triplicate and repeated all experiments three times.

### 2.10 Prediction of HOTAIR target miRNAs and miR‐17‐5p target genes

We used the starBase database (http://starbase.sysu.edu.cn/starbase2/browseNcRNA.php), version 2.0, to identify miRNAs targeted by HOTAIR; and the TargetScanHuman database (http://www.targetscan.org/vert_72/) to predict miR‐17‐5p binding sites on the target genes.

### 2.11 Transient transfection and dual luciferase reporter assay

Cells were seeded into 96‐well plates 1 day before transfection. We amplified the HOTAIR fragment and then integrated it into the pGL3 promoter carrier (Promega Corp.) to construct the report carrier wild‐type (WT)‐HOTAIR. Meanwhile, mutated fragments related to mutated (Mut) HOTAIR were cloned, and the reporting vector Mut‐HOTAIR was constructed. A total of 293T cells were transfected with 100 ng WT‐HOTAIR or Mut‐HOTAIR and 10 nmol/L miR‐17‐5p mimic/inhibitor using Lipofectamine 2000. After 48 hours of transfection, we performed an luciferase reporter assay (LRA) using a Dual‐Luciferase Reporter Assay System (Promega) as per the manufacturer’s instructions. All assays were independently performed in triplicate.

### 2.12 Western blot

Cells were harvested and lysed using ice‐cold lysis buffer (Beyotime Institute of Biotechnology), and protein concentration was determined using a bicinchoninic acid protein assay kit (Keygentec). We separated denatured proteins (20 μg) by sodium dodecyl sulphate polyacrylamide gel electrophoresis and transferred them onto polyvinylidene fluoride membranes (MilliporeSigma). After blocking, we incubated membranes at 4°C overnight with the following primary antibodies, all of which were purchased from Abcam: LC3B (1:2000; Cat. No. 192890), Beclin‐1 (1:1000; Cat. No. ab210498), ATG2 (1 µg/mL; Cat. No. ab189934), ATG7 (1:50,000; Cat. No. ab52472), and ATG16 (1:1000; Cat. No. ab187671). Subsequently, we incubated the membranes with a secondary antibody (goat anti‐rabbit; 1:10,000, Cat. No. ab205718; Abcam) for 2 hours at 25°C, visualized bound proteins via enhanced chemiluminescence (Thermo Fisher Scientific, Inc), and recorded them on an imaging system (DNR Bio‐Imaging Systems Ltd., Mahale HaHamisha). GAPDH (1:10 000, Cat. No. ab181602; Abcam) was used as the loading control.

### 2.13 Cell proliferation assay

We prepared single‐cell suspensions via trypsinization and seeded the indicated cell lines into six‐well plates at a density of 500 cells/well. After 2 weeks of culture, the cells were digested with trypsin. The 10‐μL cell suspension was mixed well with 10 μL Phenol Blue and added to the counting plate. After leaving the mixture at room temperature for 3 minutes, we observed and counted cells under an inverted microscope. The remaining cells were then inoculated in 96‐well plates at a density of 3 × 10^3^ and cultured for 24‐72 hours. We detected the optical density at 450 nm every 24 hours using a CCK‐8 kit as per the manufacturer's instructions. Each experiment was repeated three times.

### 2.14 Cell apoptosis assay

We performed this assay using an Annexin V‐Fluorescein Isothiocyanate (FITC) Apoptosis Detection Kit (Keygentec) as per the manufacturer’s instructions. Cells (10^6^ cells/mL) were harvested, washed twice with cold PBS and resuspended in 500 μL binding buffer. Subsequently, we incubated them with 5 μL Annexin V‐FITC and 5 μL propidium iodide (PI) in the dark for 15 minutes at 25°C. Cell apoptosis was assessed via flow cytometry (FCM; BD Biosciences). Each experiment was repeated three times.

### 2.15 Cell cycle assay

Cell cycle analysis was performed using the Cell Cycle Detection Kit (Keygentec). A549 and BESA2B cells (1 × 10^6^) were harvested, washed with PBS twice and fixed in 500 μL 70% ice‐cold ethanol for 2 hours at 25°C. The cells were then washed twice with cold PBS and incubated in PI (400 μL) and RNase (100 μL) for 30 minutes at 37°C in the dark. The PI signal was detected via FCM (BD Biosciences). The percentages of cells in the G1, S and G2 phase were determined and compared. Each experiment was repeated three times.

### 2.16 Statistical analysis

All data are expressed as the mean ± standard deviation (SD) and were analysed using SPSS software version 19.0 (IBM Corp.). We performed statistical analysis using one‐way analysis of variance (ANOVA) and Dunnett’s post hoc test. For independent two‐group analyses, Student’s *t* tests were used.

## 3 RESULTS

### 3.1 Discovery of ALI‐associated lncRNAs via microarray analysis

**FIGURE 1:**
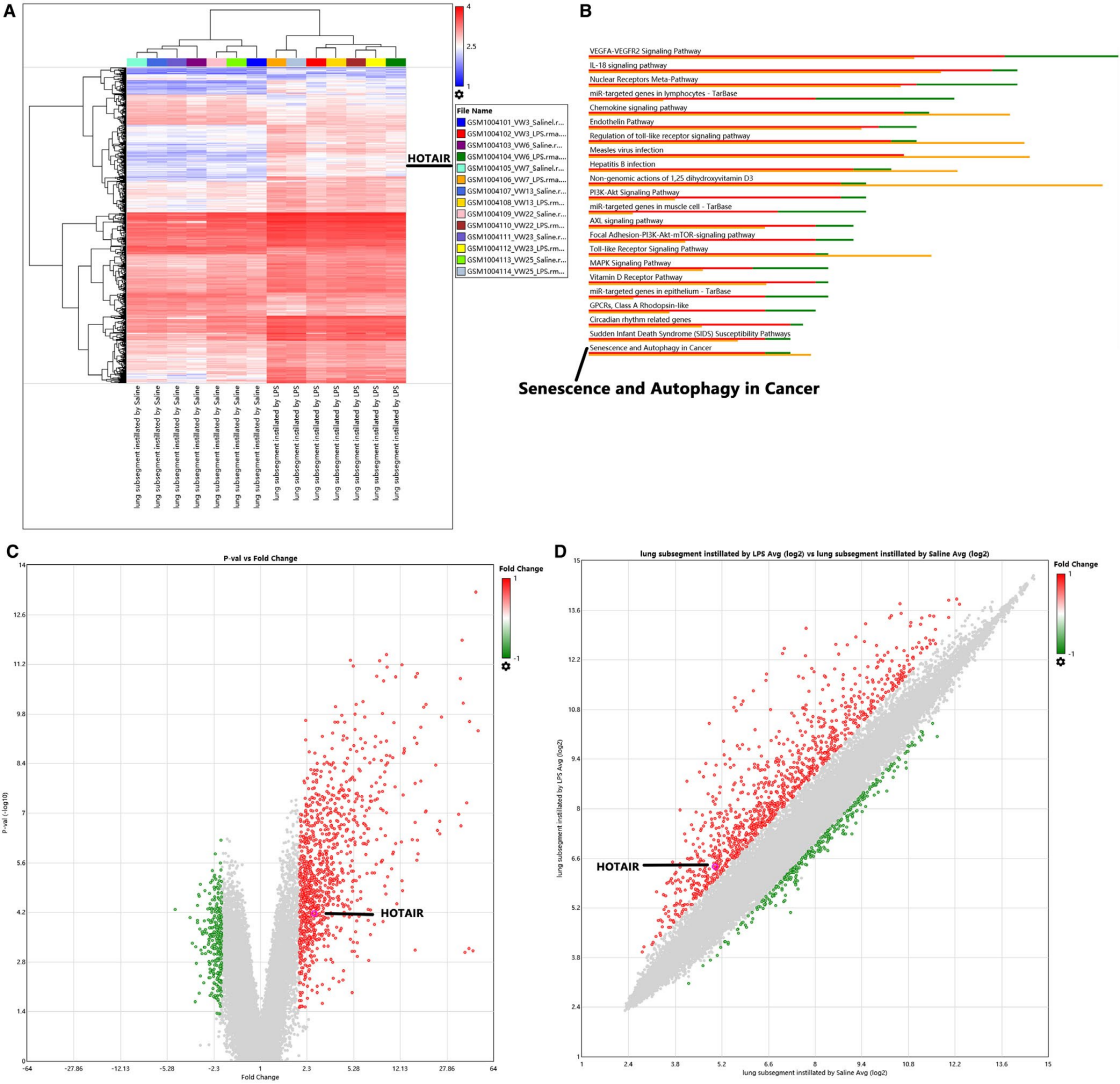
Identification of differentially expressed lncRNAs in alveolar macrophages from mouse lung subsegments instilled with LPS. A, Hierarchical‐clustering heatmap of differentially expressed lncRNAs/mRNAs in seven alveolar macrophage samples from lung subsegments instilled with LPS and seven control samples. Rows represent lncRNAs or mRNAs identified using the Affymetrix Transcriptome Analysis Console; columns represent cell specimens. Blue indicates low expression of lncRNAs/mRNAs; red indicates high expression. B, Pathway enrichment analysis of differentially expressed lncRNAs/mRNAs. The green bar indicates the number of downregulated genes; the red bar indicates the number of upregulated genes; the yellow bar indicates statistical significance. C, Volcano plot of differentially expressed lncRNAs and mRNAs. The *x*‐axis indicates fold change in lncRNA/mRNA expression between different samples; the *y*‐axis indicates statistical significance. Significantly regulated lncRNAs/mRNAs were filtered (|FC| > 2; *P* < .05) and marked by green or red dots. D, Scatter plots of differentially expressed lncRNAs and mRNAs. Significantly upregulated and downregulated lncRNAs/mRNAs are identified by green and red dots, respectively

We investigated the differential expression of lncRNAs/mRNAs in alveolar macrophages from lung subsegments instilled with LPS using raw microarray data obtained from the NCBI GEO database (GSE40885). Of the 1289 lncRNAs/mRNAs detected via microarray analysis, 1011 were upregulated in alveolar macrophages from LPS‐instilled lung subsegments compared to controls when using the criteria of mean |FC| > 2 and *P* <.05 (Figure 1B,C). HOTAIR, LINC01093, LINC01215, LINC01268 and LINC00189 were among the significantly upregulated ncRNAs in alveolar macrophages from LPS‐instilled lung subsegments (Figure 1A). Among them, lncRNA HOTAIR as an oncogene has been confirmed by numerous studies and plays a key role in tumour development.^21^, ^22^ However, the role and molecular mechanism of lncRNA HOTAIR in ALI have not been reported yet. Therefore, we chose HOTAIR gene as the target lncRNA of this study for further discussion.

In addition, we identified the top 23 pathways associated with these differentially expressed lncRNAs via KEGG pathway analysis. Of these 23, the most significantly enriched and relevant were the interleukin‐18 (IL‐18) signalling pathway as well as the senescence and autophagy in cancer signalling pathway, with the latter corresponding to 14 upregulated genes.

### 3.2 HOTAIR regulated the proliferation, apoptosis, cell cycle progression and autophagy of LPS‐induced A549 and BEAS‐2B cells

**FIGURE 2:**
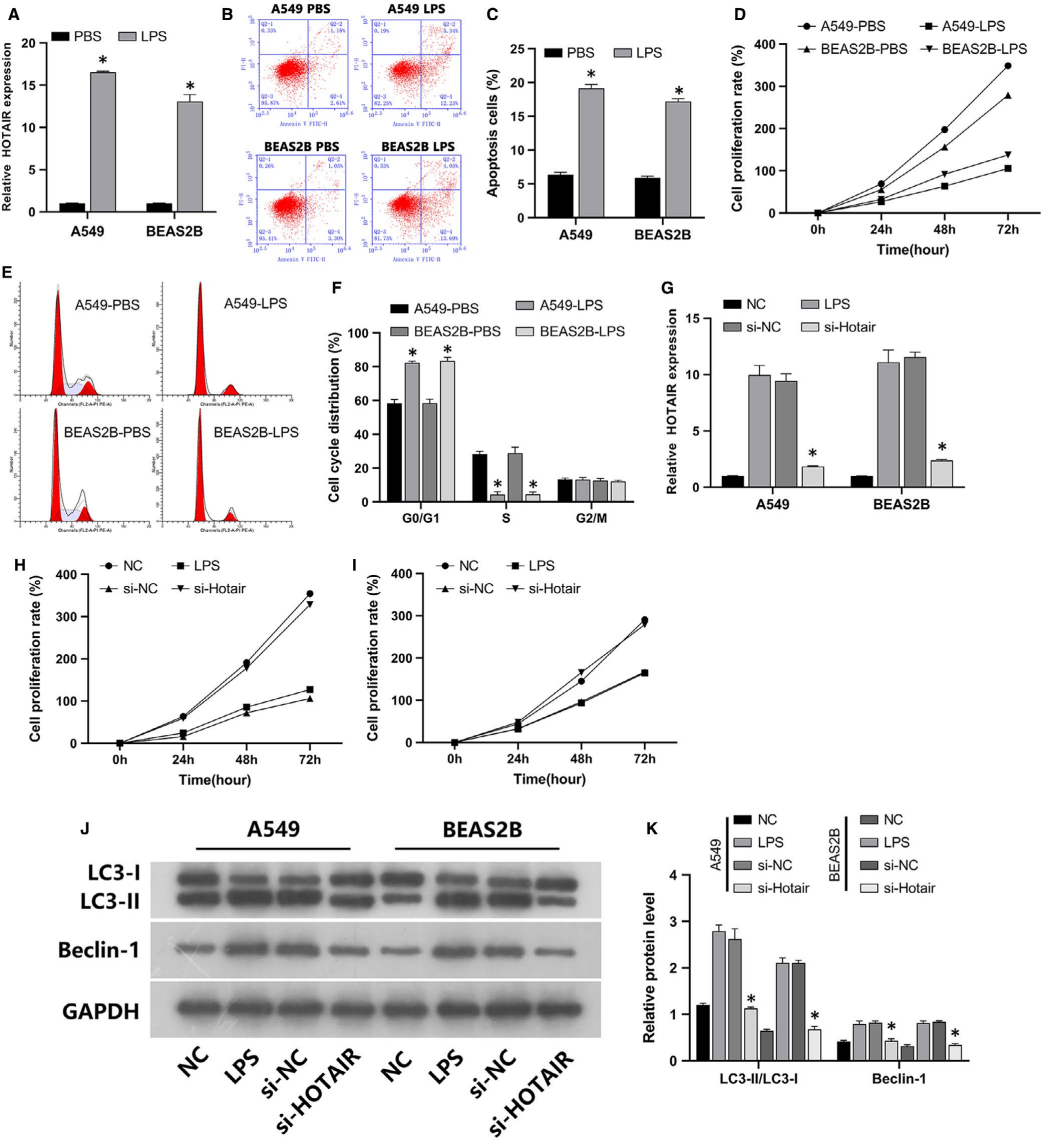
The lncRNA HOTAIR regulated autophagy, apoptosis, cell cycle progression, and the proliferation of LPS‐stimulated A549 and BEAS‐2B cells. A, Results of qRT‐PCR analysis. The black bar represents HOTAIR expression in the PBS treatment group; the grey bar represents HOTAIR expression in the LPS treatment group; error bars show 95% confidence intervals. B, Representative FCM images indicate that LPS‐stimulated A549 and BEAS‐2B cells underwent increased apoptosis. C, The quantitative column figure represents the percentage of apoptotic cells (mean ± SD) from three independent experiments. D, Cell proliferation was determined via CCK‐8 assays at 24, 48 and 72 hours. A549 and BEAS‐2B cells treated with PBS proliferated faster than those treated with LPS. E, Representative FCM images indicated that LPS‐treated A549 and BEAS‐2B cells underwent G0/G1 phase arrest. F, The quantitative column figure represents the percentage of cell per cell cycle stage (mean ± SD) from three independent experiments. G, The effects of NC, LPS, si‐NC and si‐HOTAIR on HOTAIR expression were assessed in A549 and BEAS‐2B cells via qRT‐PCR. H,I, The effects of NC, LPS, si‐NC and si‐HOTAIR on cell proliferation were assessed in A549 and BEAS‐2B cells via CCK‐8 assays. J,K, The effects of NC, LPS, si‐NC and si‐HOTAIR on the expression of autophagy marker proteins LC3B and Beclin‐1 in A549 and BEAS‐2B cells were assessed via WB. *P < .05

Using RT‐qPCR, we confirmed HOTAIR upregulation in LPS‐induced A549 and BEAS‐2B cells (Figure 2A). The expression pattern of HOTAIR in these cells was consistent with data obtained via microarray transcriptome analysis. HOTAIR was then subjected to further analysis. Our observations were consistent with those of previous studies reporting that LPS‐induced A549 and BEAS‐2B cells can be used as ALI cell models.^3^, ^4^ LPS increased apoptosis (Figure 2B,C) and decreased proliferation (Figure 2D), arresting cells in the G0/G1 phase (Figure 2E,F). Therefore, we next explored the effects of HOTAIR in these ALI cell models.

**FIGURE 3:**
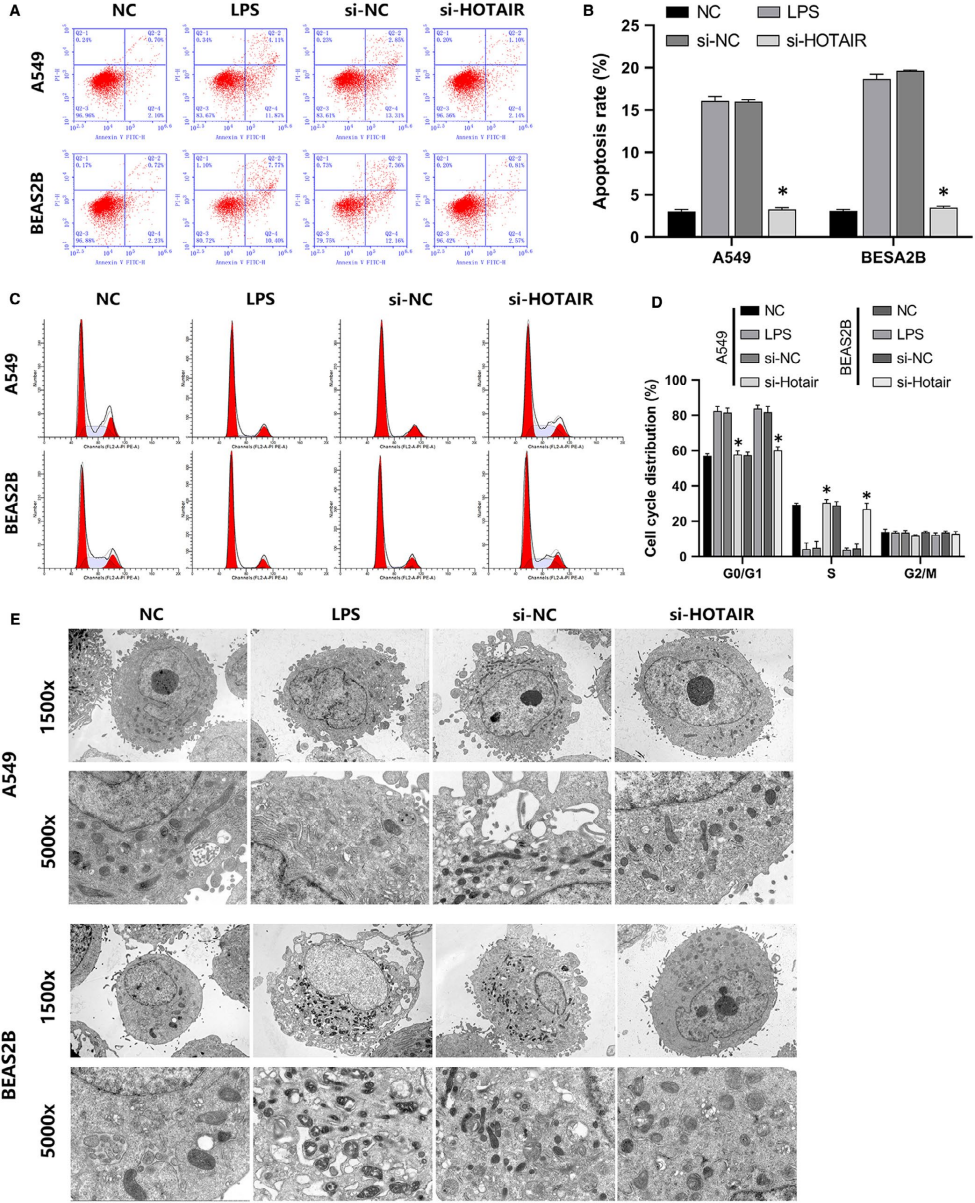
HOTAIR regulated the cell cycle, apoptosis and autophagy of A549 and BEAS‐2B cells. A, FCM analysis indicated apoptosis in the NC, LPS, si‐NC and si‐HOTAIR groups. B, The quantitative column figure represents the percentage of apoptotic cells (mean ± SD) from three independent experiments. C, FCM was used to determine cell cycle progression in the NC, LPS, si‐NC and si‐HOTAIR groups. D, The quantitative column figure represents the percentage of cells in each cycle stage (mean ± SD) from three independent experiments. E, The effects of NC, LPS, si‐NC, and si‐HOTAIR on autophagosome formation were assessed in A549 and BEAS‐2B cells via TEM. *P < .05

RT‐qPCR confirmed that si‐NC and si‐HOTAIR were efficiently transfected into LPS‐stimulated A549 and BEAS‐2B cells. LPS enhanced HOTAIR expression, but this was reversed by si‐HOTAIR transfection (Figure 2G). CCK‐8 results indicated that LPS reduced A549 and BEAS‐2B cell proliferation, which was also reversed via si‐HOTAIR (Figure 2H,I). Western blot (WB) revealed that LPS induced the expression of the autophagy marker proteins LC3B and Beclin‐1, but si‐HOTAIR suppressed this effect as well (Figure 2J,K). FCM results indicated that LPS increased the apoptosis (Figure 3A,B) and G0/G1 phase arrest (Figure 3C,D) of A549 as well as BEAS‐2B cells, which was also reversed via si‐HOTAIR transfection. TEM revealed that LPS increased autophagic vacuoles. Once again, si‐HOTAIR reversed this phenomenon in both A549 and BEAS‐2B cells (Figure 3E). Taken together, these results suggested that inhibition of HOTAIR suppressed LPS‐induced A549 and BEAS‐2B cell autophagy, apoptosis and cycle arrest.

### 3.3 MiR‐17‐5p inhibition reversed the effects of si‐HOTAIR on cell autophagy, proliferation and apoptosis

**FIGURE 4:**
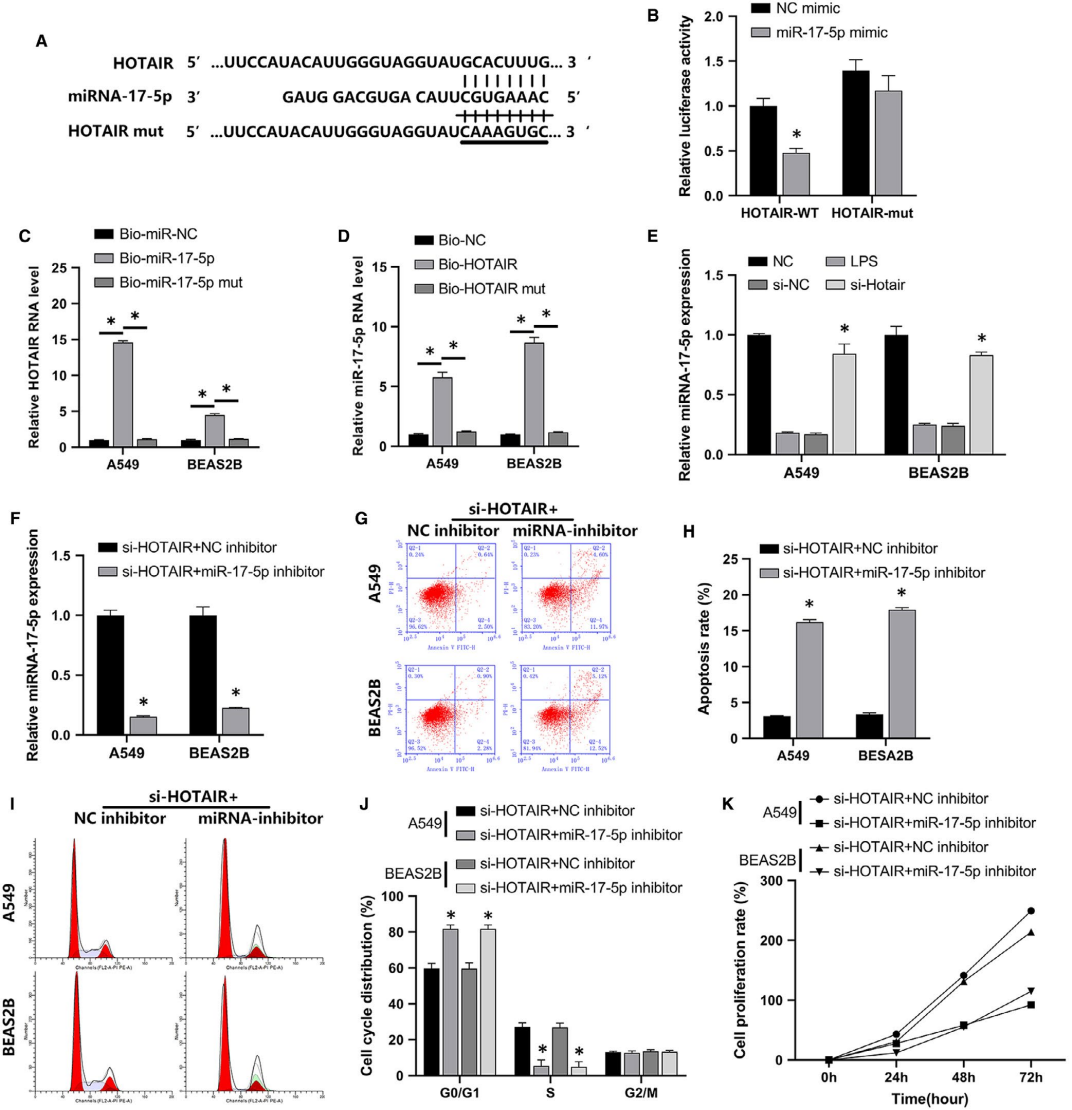
MiR‐17‐5p reversed HOTAIR‐mediated LPS‐induced changes in A549 and BEAS‐2B cell function. A, LncRNA HOTAIR‐miR‐17‐5p binding sites predicted using the starBase database. B, A dual LRA was employed to validate the binding of miR‐17‐5p/HOTAIR. C, D, RNA pull‐down validated the binding of lncRNA HOTAIR and miR‐17‐5p. E, Effects of NC, LPS, si‐NC and si‐HOTAIR on miR‐17‐5p expression were assessed in A549 and BEAS‐2B cells via qRT‐PCR. F, The effects of co‐transfection with si‐HOTAIR/NC inhibitor or si‐HOTAIR/miR‐17‐5p were assessed in A549 and BEAS‐2B cells via qRT‐PCR. G, FCM was used to detect apoptosis in the si‐HOTAIR +NC inhibitor group and the si‐HOTAIR +miR‐17‐5p inhibitor group. H, The quantitative column figure represents the percentage of apoptotic cells (mean ± SD) from three independent experiments. I, FCM was used to detect cell cycle in the si‐HOTAIR +NC inhibitor group and the si‐HOTAIR +miR‐17‐5p inhibitor group. J, The quantitative column figure represents the cells in each cycle stage (mean ± SD) from three independent experiments. K, The effect of co‐transfection on cell proliferation was assessed in A549 and BEAS‐2B cells via CCK‐8 assays. *P < .05

Using the starBase online database, we found that miR‐17‐5p was a potential target of HOTAIR (Figure 4A). Dual LRA results confirmed that miR‐17‐5p directly interacted with HOTAIR (Figure 4B). In order to verify the miR‐17‐5p‐HOTAIR interaction, RNA pull‐down was employed. The results indicated that HOTAIR and miR‐17‐5p RNA level were significantly higher in A549 and BEAS‐2B cells compared to control and Mut groups (Figure 4C,D). Furthermore, RT‐qPCR results confirmed that LPS reduced miR‐17‐5p expression and that si‐HOTAIR reversed this reduction in A549 and BEAS‐2B cells (Figure 4E).

**FIGURE 5:**
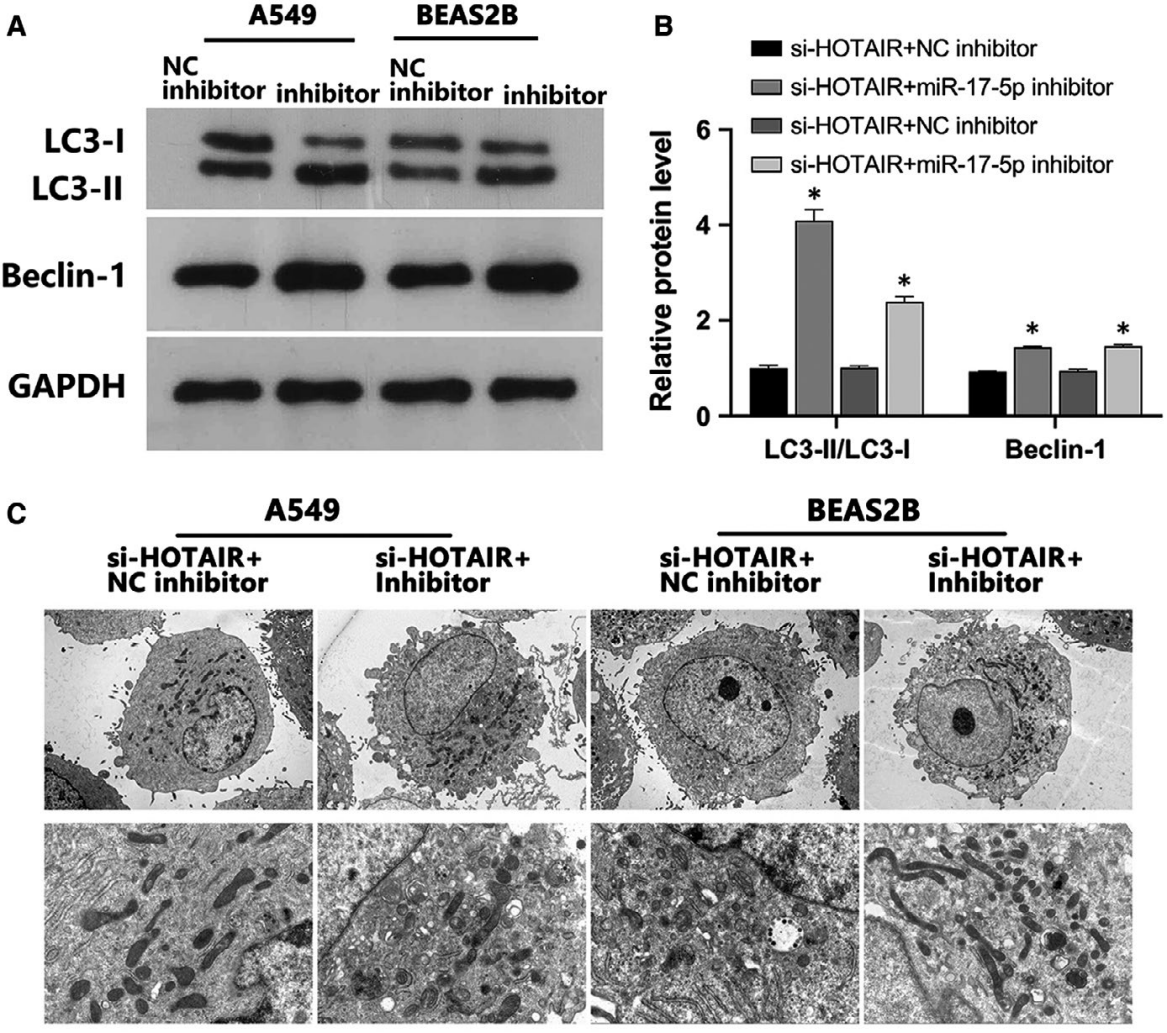
MiR‐17‐5p inhibitor reversed HOTAIR‐mediated autophagy in A549 and BEAS‐2B cells. A, B, The effect of co‐transfection on the expression of autophagy marker proteins LC3B and Beclin‐1 was assessed in A549 and BEAS‐2B cells via WB. C, TEM observation of autophagy vacuoles in A549 and BEAS‐2B cells of the si‐HOTAIR +NC inhibitor and si‐HOTAIR +miR‐17‐5p inhibitor groups. *P < .05

Subsequently, we performed co‐transfection experiments with si‐HOTAIR/NC inhibitor or si‐HOTAIR/miR‐17‐5p inhibitor to determine the effect on miR‐17‐5p expression in A549 and BEAS‐2B cells. RT‐qPCR further confirmed LRA results (Figure 4F). FCM indicated that miR‐17‐5p inhibition increased apoptosis (Figure 4G,H) and arrested LPS‐induced A549 and BEAS‐2B cells in the G0/G1 phase prior to their entry into S phase (Figure 4I,J) after co‐transfection with si‐HOTAIR compared to the NC inhibitor. Further, the si‐HOTAIR‐mediated increase in cell proliferation was reversed in the miR‐17‐5p inhibitor group compared with the NC inhibitor group (Figure 4K). The protein expression of autophagy markers LC3B and Beclin‐1 was increased after co‐transfection with si‐HOTAIR and miR‐17‐5p inhibitor compared with the NC inhibitor group (Figure 5A,B). Similarly, less LPS‐induced autophagy vacuoles were observed under NC co‐transfection, whereas miR‐17‐5p inhibitor co‐transfection had the opposite effect (Figure 5C). Altogether, miR‐17‐5p inhibition counteracted the si‐HOTAIR‐mediated suppression of autophagy, apoptosis, cell cycle progression and proliferation of A549 and BEAS‐2B cells.

### 3.4 MiR‐17‐5p inhibition reversed the si‐HOTAIR‐mediated reduction in ATG expression

**FIGURE 6:**
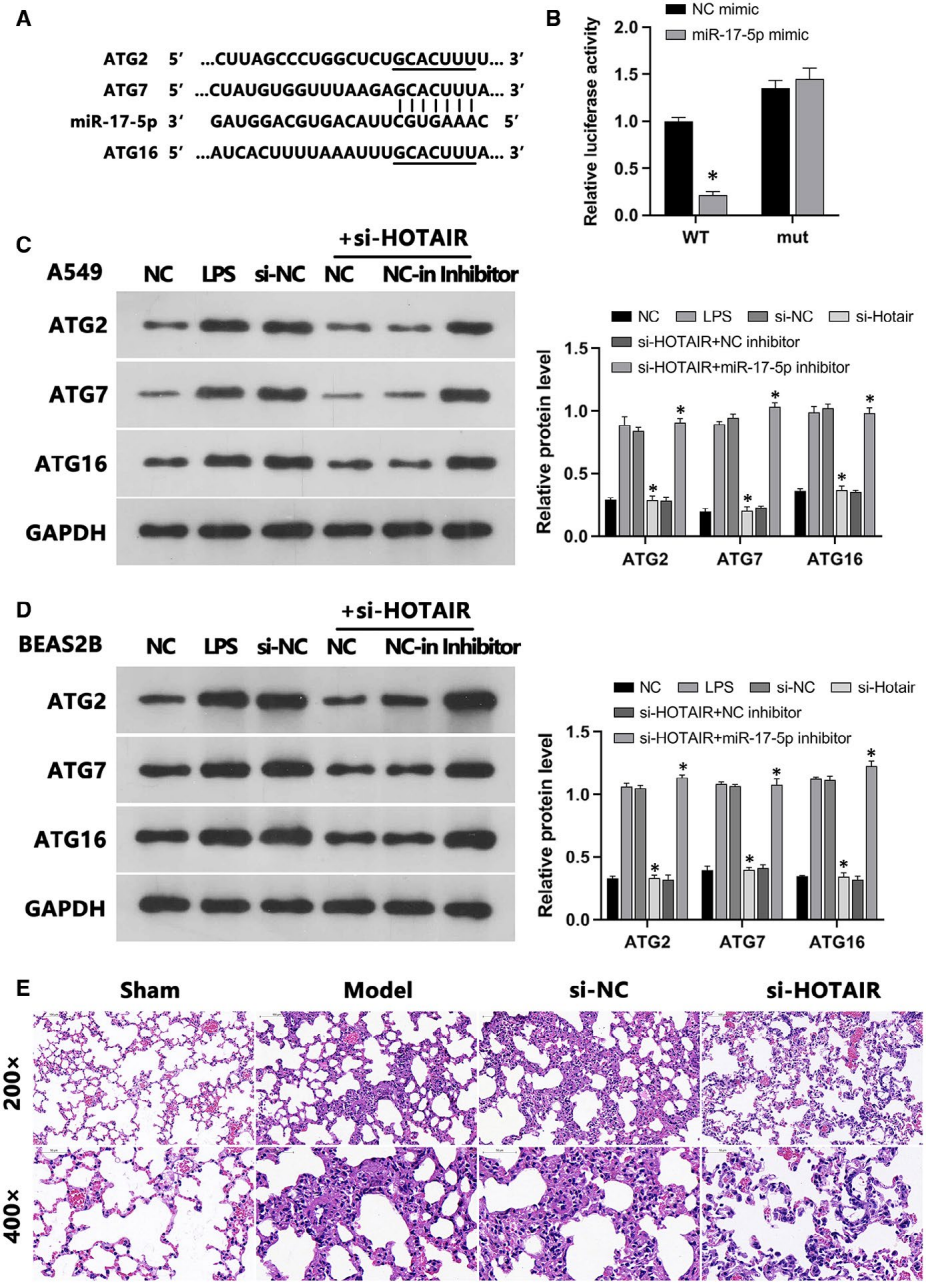
MiR‐17‐5p reversed the HOTAIR‐mediated increase in the expression of autophagy‐related proteins ATG2, ATG7 and ATG16 in LPS‐induced A549 and BEAS‐2B cells. A, Binding sites of miR‐17‐5p and ATG2/ATG7/ATG16 were predicted. B, Dual LRA was used to validate the binding of miR‐17‐5p/ATG2/ATG7/ATG16. C, D, The effects of NC, LPS, si‐NC, si‐HOTAIR, si‐HOTAIR/NC inhibitor and si‐HOTAIR/miR‐17‐5p inhibitor on the expression of ATG2, ATG7 and ATG16 in A549 and BEAS‐2B cells were assessed via WB. E, Inhibition of HOTAIR reduced ALI in vivo. Morphology of lung tissues from C57/6 mice was assessed via H&E staining, whereas expression of HOTAIR in these tissues was determined via qRT‐PCR. Groups included the sham operation group, ALI model group, LV‐Control group and LV‐si‐HOTAIR group. *P < .05

Using the TargetScanHuman online database, we found that ATGs *ATG2*, *ATG7* and *ATG16* were potential targets of miR‐17‐5p (Figure 6A), which was confirmed by dual LRA (Figure 6B). Subsequently, WB indicated that their protein expression was enhanced by LPS, whereas si‐HOTAIR transfection reversed this effect. Meanwhile, co‐transfection of si‐HOTAIR and NC inhibitor suppressed ATG2, ATG7, ATG16 protein levels, whereas co‐transfection with miR‐17‐5p inhibitor led to the opposite outcome (Figure 6C,D).

### 3.5 Si‐HOTAIR significantly reversed LPS‐induced ALI in vivo

Lung tissue reflects the severity of LPS‐induced ALI in mice. After H&E staining, we observed that the lung tissue of mice in the sham group exhibited normal structure, intact alveolar walls, no obvious oedema in the alveolae and lung interstitium, as well as no inflammatory cell infiltration. Compared with sham group mice, those in the model and LV‐control groups had significant pulmonary oedema, tissue damage and alveolar septal thickening. Further, when compared to mice of the LV‐Control group, the alveolar septa of LV‐si‐HOTAIR mice were significantly thinner without obvious oedema and tissue damage as well as reduced inflammatory cell infiltration (Figure 6E). HOTAIR expression was significantly lower in the LV‐si‐HOTAIR group compared to the LV‐control group. These results suggested that inhibition of HOTAIR alleviated LPS‐induced ALI in mice.

## 4 DISCUSSION

HOTAIR, a cell cycle‐associated lncRNA, is linked to a range of major diseases, including cancer.^23^, ^24^ Studies have shown that lncRNA is closely related to cellular functions such as proliferation and apoptosis as well as cancer cell migration and invasion.^25^, ^26^, ^27^ However, the underlying mechanisms of HOTAIR in ALI remain poorly understood. A key aspect of the current study is that we provided a comprehensive functional and mechanistic characterization of HOTAIR in LPS‐induced ALI. We identified HOTAIR via microarray data mining, confirming its upregulation in LPS‐induced ALI cell models. In A549 and BEAS‐2B cells, HOTAIR inhibition suppressed LPS‐induced apoptosis, whereas cell proliferation, S phase entry and DNA synthesis were promoted. Similarly, si‐HOTAIR reversed the LPS‐induced ALI effects in vivo, which contrasted with the role of lncRNA NEAT1 in ALI.^20^ These findings highlight the potential of HOTAIR as a therapeutic target in ALI. We also observed an association between HOTAIR and autophagy. In order to improve the reliability of our results, we employed two validation standards for autophagy, namely the number of autophagosomes observed via TEM and the expression of the autophagy marker proteins LC3B and Beclin‐1. Further, HOTAIR mediated the upregulation in ATG2, ATG7 and ATG16 expression in LPS‐stimulated A549 and BEAS‐2B cells, highlighting the need for further research into HOTAIR‐regulated autophagy in the context of ALI.

MiR‐17‐5p, a key regulator of the G1/S cell cycle transition, is implicated in various diseases.^28^, ^29^ Chen *et al* ^30^ reported that downregulation of miR‐17‐5p aggravated brain damage, whereas Zhao *et al* ^31^ found that miR‐17‐5p contributes to tumour growth and metastasis in gastric cancer. In the current study, we observed that miR‐17‐5p induced the proliferation of LPS‐stimulated A549 and BEAS‐2B cells, playing a similar role in ALI. Furthermore, miR‐17‐5p was previously reported to target HOTAIR, thus promoting osteoarthritis progression,^32^ and provides a reliable basis for us to study the interaction of HOTAIR/miR‐17‐5p. Using the starBase database, we predicted that miR‐17‐5p targeted HOTAIR, which we subsequently confirmed via dual LRA, RNA pull‐down RT‐qPCR, cell function assays. Thus, miR‐17‐5p was suggested as a novel therapeutic target in ALI worthy of further investigation. Interestingly, previous studies reported that miR‐17‐5p regulates autophagy in several diseases.^33^, ^34^ Bobbili *et al* ^35^ described miR‐17‐5p as an essential regulator of autophagy and an ‘alarm signal’ in cancer. We observed a negative association between miR‐17‐5p and autophagy, as si‐HOTAIR suppressed autophagosome formation in LPS‐stimulated A549 and BEAS‐2B cells, whereas miR‐17‐5p inhibition upregulated ATG2, ATG7, and ATG16 protein expression. Taken together, these findings indicated that HOTAIR induces autophagy in ALI by adsorbing miR‐17‐5p.

Acute lung injury is a severe respiratory disorder associated with acute as well as persistent lung inflammation.^36^ The role of autophagy in ALI has attracted increasing attention among researchers.^37^, ^38^ For instance, Hu *et al* ^39^ found that complement component 5a (C5a) aggravated ALI via the autophagy‐mediated apoptosis of alveolar macrophages. In the current study, we found that autophagy was regulated to affect the pathological progression of ALI as well as ATG2, ATG7 and ATG16 expression. Filfan *et al* ^40^ reported that the autophagy‐related proteins Beclin‐1, LC3, ATG5 and ATG7 are associated with ALI. In our study, the upregulation of ATG2, ATG7 and ATG16 occurred in parallel to the inhibition of LPS‐stimulated A549 and BEAS‐2B cell proliferation. These findings highlighted the therapeutic potential of afore‐mentioned ATGs as drug targets.

In conclusion, our findings elucidate the molecular mechanisms of HOTAIR underlying ALI. We confirmed the upregulation of HOTAIR in LPS‐induced ALI cell models. Further, functional experiments indicated that HOTAIR affected autophagy, apoptosis, the cell cycle and proliferation by regulating miR‐17‐5p, highlighting the therapeutic relevance of this signalling axis in LPS‐induced ALI.

## CONFLICT OF INTEREST

The authors confirm that there are no conflicts of interest.

## AUTHOR CONTRIBUTIONS

**Yujun Li:** Conceptualization (supporting); Data curation (equal); Formal analysis (equal); Investigation (supporting); Methodology (equal); Project administration (supporting); Validation (equal); Visualization (equal); Writing‐original draft (equal); Writing‐review & editing (equal). **Zhike Liang:** Data curation (equal); Formal analysis (equal); Methodology (equal); Writing‐original draft (equal); Writing‐review & editing (equal). **Hua He:** Data curation (supporting); Methodology (supporting); Software (supporting); Visualization (supporting). **Xiaomei Huang:** Data curation (supporting); Methodology (supporting); Software (supporting); Visualization (supporting). **Zexun Mo:** Data curation (equal); Methodology (equal); Software (supporting); Visualization (supporting). **Jinwen Tan:** Data curation (supporting); Methodology (supporting); Software (supporting); Visualization (supporting). **Weihong Guo:** Data curation (supporting); Methodology (supporting); Software (supporting); Visualization (supporting). **Ziwen Zhao:** Conceptualization (equal); Investigation (equal); Project administration (equal); Resources (equal); Supervision (equal). **Shuquan Wei:** Conceptualization (lead); Funding acquisition (lead); Investigation (equal); Project administration (lead); Resources (lead); Software (lead); Supervision (lead).

## Data Availability

The datasets used and/or analysed during the current study are available from the corresponding author on reasonable request.

## References

[1] Zhang Y, Li X, Grailer JJ, et al. Melatonin alleviates acute lung injury through inhibiting the NLRP3 inflammasome. J Pineal Res. 2016;60:405‐414. 26888116 10.1111/jpi.12322

[2] Han J, Li H, Bhandari S, et al. Maresin conjugates in tissue regeneration 1 improves alveolar fluid clearance by up‐regulating alveolar ENaC, Na, K‐ATPase in lipopolysaccharide‐induced acute lung injury. J Cell Mol Med. 2020;24:4736‐4747. 32160403 10.1111/jcmm.15146 PMC7176857

[3] He YQ, Zhou CC, Yu LY, et al. Natural product derived phytochemicals in managing acute lung injury by multiple mechanisms. Pharmacol Res. 2020;163:105224. 33007416 10.1016/j.phrs.2020.105224 PMC7522693

[4] Li H, Shi H, Gao M, Ma N, Sun R. Long non‐coding RNA CASC2 improved acute lung injury by regulating miR‐144‐3p/AQP1 axis to reduce lung epithelial cell apoptosis. Cell Biosci. 2018;8:15. 29492259 10.1186/s13578-018-0205-7 PMC5828141

[5] Gustafsson AB, Dorn GW 2nd. Evolving and expanding the roles of mitophagy as a homeostatic and pathogenic process. Physiol Rev. 2019;99:853‐892. 30540226 10.1152/physrev.00005.2018 PMC6442924

[6] Lenoir O, Jasiek M, Henique C, et al. Endothelial cell and podocyte autophagy synergistically protect from diabetes‐induced glomerulosclerosis. Autophagy. 2015;11:1130‐1145. 26039325 10.1080/15548627.2015.1049799 PMC4590611

[7] Fu Z, Zhang Z, Wu X, Zhang J. Hydrogen‐rich saline inhibits lipopolysaccharide‐induced acute lung injury and endothelial dysfunction by regulating autophagy through mTOR/TFEB signaling pathway. Biomed Res Int. 2020;2020:9121894. 32071922 10.1155/2020/9121894 PMC7011387

[8] Chen WX, Zhou J, Zhou SS, et al. Microvesicles derived from human Wharton's jelly mesenchymal stem cells enhance autophagy and ameliorate acute lung injury via delivery of miR‐100. Stem Cell Res Ther. 2020;11:113. 32169098 10.1186/s13287-020-01617-7 PMC7071666

[9] Yang A, Bofill‐De Ros X, Shao TJ, et al. 3' Uridylation confers miRNAs with non‐canonical target repertoires. Mol Cell. 2019;75:511‐522.e4. 31178353 10.1016/j.molcel.2019.05.014 PMC6688926

[10] Spadotto V, Giambruno R, Massignani E, et al. PRMT1‐mediated methylation of the microprocessor‐associated proteins regulates microRNA biogenesis. Nucleic Acids Res. 2020;48:96‐115. 31777917 10.1093/nar/gkz1051 PMC6943135

[11] Rajasekaran S, Pattarayan D, Rajaguru P, Sudhakar Gandhi PS, Thimmulappa RK. MicroRNA regulation of acute lung injury and acute respiratory distress syndrome. J Cell Physiol. 2016;231:2097‐2106. 26790856 10.1002/jcp.25316

[12] Jansing JC, Fiedler J, Pich A, et al. miR‐21‐KO alleviates alveolar structural remodeling and inflammatory signaling in acute lung injury. Int J Mol Sci. 2020;21. 10.3390/ijms21030822 PMC7037600 32012801

[13] Neudecker V, Brodsky KS, Clambey ET, et al. Neutrophil transfer of miR‐223 to lung epithelial cells dampens acute lung injury in mice. Sci Transl Med. 2017;9:eaah5360. 28931657 10.1126/scitranslmed.aah5360 PMC5842431

[14] Zhou Z, You Z. Mesenchymal stem cells alleviate LPS‐induced acute lung injury in mice by MiR‐142a‐5p‐controlled pulmonary endothelial cell autophagy. Cell Physiol Biochem. 2016;38:258‐266. 26784440 10.1159/000438627

[15] Sherpa C, Rausch JW, Le Grice SF. Structural characterization of maternally expressed gene 3 RNA reveals conserved motifs and potential sites of interaction with polycomb repressive complex 2. Nucleic Acids Res. 2018;46:10432‐10447. 30102382 10.1093/nar/gky722 PMC6212721

[16] Zhang J, Wang P, Wan L, Xu S, Pang D. The emergence of noncoding RNAs as Heracles in autophagy. Autophagy. 2017;13:1004‐1024. 28441084 10.1080/15548627.2017.1312041 PMC5486373

[17] Wang J, Shen YC, Chen ZN, et al. Microarray profiling of lung long non‐coding RNAs and mRNAs in lipopolysaccharide‐induced acute lung injury mouse model. Biosci Rep. 2019;39:BSR20181634. 30979832 10.1042/BSR20181634 PMC6488857

[18] Liao H, Zhang S, Qiao J. Silencing of long non‐coding RNA MEG3 alleviates lipopolysaccharide‐induced acute lung injury by acting as a molecular sponge of microRNA‐7b to modulate NLRP3. Aging. 2020;12:20198–20211. 32852284 10.18632/aging.103752 PMC7655187

[19] Liu M, Han T, Shi S, Chen E. Long noncoding RNA HAGLROS regulates cell apoptosis and autophagy in lipopolysaccharides‐induced WI‐38cells via modulating miR‐100/NF‐kappaB axis. Biochem Biophys Res Commun. 2018;500:589‐596. 29673591 10.1016/j.bbrc.2018.04.109

[20] Chen C, Zhang H, Ge M, Ye J, Li R, Wang D. LncRNA NEAT1 acts as a key regulator of cell apoptosis and inflammatory response by the miR‐944/TRIM37 axis in acute lung injury. J Pharmacol Sci. 2021;145:202‐212. 33451755 10.1016/j.jphs.2020.11.009

[21] Rajagopal T, Talluri S, Akshaya RL, Dunna NR. HOTAIR LncRNA: a novel oncogenic propellant in human cancer. Clin Chim Acta. 2020;503:1‐18. 31901481 10.1016/j.cca.2019.12.028

[22] Chang L, Guo R, Yuan Z, Shi H, Zhang D. LncRNA HOTAIR regulates CCND1 and CCND2 expression by sponging miR‐206 in ovarian cancer. Cell Physiol Biochem. 2018;49:1289‐1303. 30205383 10.1159/000493408

[23] Loewen G, Jayawickramarajah J, Zhuo Y, Shan B. Functions of lncRNA HOTAIR in lung cancer. J Hematol Oncol. 2014;7:90. 25491133 10.1186/s13045-014-0090-4 PMC4266198

[24] Zhang JX, Han L, Bao ZS, et al. Chinese Glioma Cooperative G. HOTAIR, a cell cycle‐associated long noncoding RNA and a strong predictor of survival, is preferentially expressed in classical and mesenchymal glioma. Neuro Oncol. 2013;15:1595‐1603. 24203894 10.1093/neuonc/not131 PMC3829598

[25] Zhao W, Geng D, Li S, Chen Z, Sun M. LncRNA HOTAIR influences cell growth, migration, invasion, and apoptosis via the miR‐20a‐5p/HMGA2 axis in breast cancer. Cancer Med. 2018;7:842‐855. 29473328 10.1002/cam4.1353 PMC5852357

[26] Liu XH, Sun M, Nie FQ, et al. Lnc RNA HOTAIR functions as a competing endogenous RNA to regulate HER2 expression by sponging miR‐331‐3p in gastric cancer. Mol Cancer. 2014;13:92. 24775712 10.1186/1476-4598-13-92 PMC4021402

[27] Zhan S, Wang K, Song Y, et al. Long non‐coding RNA HOTAIR modulates intervertebral disc degenerative changes via Wnt/beta‐catenin pathway. Arthritis Res Ther. 2019;21:201. 31481088 10.1186/s13075-019-1986-8 PMC6724301

[28] Cloonan N, Brown MK, Steptoe AL, et al. The miR‐17‐5p microRNA is a key regulator of the G1/S phase cell cycle transition. Genome Biol. 2008;9:R127. 18700987 10.1186/gb-2008-9-8-r127 PMC2575517

[29] Cheng YY, Wright CM, Kirschner MB, et al. KCa1.1, a calcium‐activated potassium channel subunit alpha 1, is targeted by miR‐17‐5p and modulates cell migration in malignant pleural mesothelioma. Mol Cancer. 2016;15:44. 27245839 10.1186/s12943-016-0529-z PMC4888473

[30] Chen D, Dixon BJ, Doycheva DM, et al. IRE1alpha inhibition decreased TXNIP/NLRP3 inflammasome activation through miR‐17‐5p after neonatal hypoxic‐ischemic brain injury in rats. J Neuroinflammation. 2018;15:32. 29394934 10.1186/s12974-018-1077-9 PMC5797348

[31] Zhao J, Xiao A, Liu C, et al. The HIF‐1A/miR‐17‐5p/PDCD4 axis contributes to the tumor growth and metastasis of gastric cancer. Signal Transduct Target Ther. 2020;5:46. 32296039 10.1038/s41392-020-0132-z PMC7145839

[32] Hu J, Wang Z, Shan Y, Pan Y, Ma J, Jia L. Long non‐coding RNA HOTAIR promotes osteoarthritis progression via miR‐17‐5p/FUT2/beta‐catenin axis. Cell Death Dis. 2018;9:711. 29907764 10.1038/s41419-018-0746-z PMC6003907

[33] Pang F, Liu C, Cui Y, et al. miR‐17‐5p promotes proliferation and migration of CAL‐27 human tongue squamous cell carcinoma cells involved in autophagy inhibition under hypoxia. Int J Clin Exp Pathol. 2019;12:2084‐2091. 31934030 PMC6949637

[34] Shi YP, Liu GL, Li S, Liu XL. miR‐17‐5p knockdown inhibits proliferation, autophagy and promotes apoptosis in thyroid cancer via targeting PTEN. Neoplasma. 2020;67:249‐258. 31973533 10.4149/neo_2019_190110N29

[35] Bobbili MR, Mader RM, Grillari J, Dellago H. OncomiR‐17‐5p: alarm signal in cancer? Oncotarget. 2017;8:71206‐71222. 29050357 10.18632/oncotarget.19331 PMC5642632

[36] Lee BW, Ha JH, Shin HG, et al. Spiraea prunifolia var. simpliciflora Attenuates Oxidative Stress and Inflammatory Responses in a Murine Model of Lipopolysaccharide‐Induced Acute Lung Injury and TNF‐alpha‐Stimulated NCI‐H292 Cells. Antioxidants (Basel). 2020;9. 10.3390/antiox9030198 PMC7139931 32111036

[37] Cabrera S, Maciel M, Herrera I, et al. Essential role for the ATG4B protease and autophagy in bleomycin‐induced pulmonary fibrosis. Autophagy. 2015;11:670‐684. 25906080 10.1080/15548627.2015.1034409 PMC4502665

[38] Domigan CK, Warren CM, Antanesian V, et al. Autocrine VEGF maintains endothelial survival through regulation of metabolism and autophagy. J Cell Sci. 2015;128:2236‐2248. 25956888 10.1242/jcs.163774 PMC4487014

[39] Hu R, Chen ZF, Yan J, et al. Complement C5a exacerbates acute lung injury induced through autophagy‐mediated alveolar macrophage apoptosis. Cell Death Dis. 2014;5:e1330. 25032853 10.1038/cddis.2014.274 PMC4123068

[40] Filfan M, Sandu RE, Zavaleanu AD, et al. Autophagy in aging and disease. Rom J Morphol Embryol. 2017;58:27‐31. 28523294

